# The First Death in Japan Attributed to COVID-19: A Brief Report

**DOI:** 10.7759/cureus.20721

**Published:** 2021-12-26

**Authors:** Kisako Nagayama, Kentaro Watai, Kiyoshi Sekiya, Yosuke Kamide, Goro Kaneda

**Affiliations:** 1 Clinical Research Center for Allergy and Rheumatology, National Hospital Organization Sagamihara National Hospital, Kanagawa, JPN; 2 Course of Allergy and Clinical Immunology, Juntendo University Graduate School of Medicine, Tokyo, JPN; 3 Surgery, National Hospital Organization Sagamihara National Hospital, Kanagawa, JPN

**Keywords:** covid-19 pneumonia, negative pressure chamber, nosocomial infection, covid-19, acute respiratory distress syndrome [ards]

## Abstract

Our hospital encountered the first coronavirus disease 19 (COVID-19) pneumonia death in Japan. Moreover, we prevented nosocomial infection by taking appropriate infection control measures, without a negative pressure chamber. The patient was an 82-year-old woman who had no history of traveling to Wuhan or any direct contact with individuals who had been to Wuhan. Our patient had a seven-day history of fatigue, sudden fever, and hypoxemia. Chest computerized tomography images revealed peripheral ground-glass opacities in her lungs. A diagnostic COVID-19 reverse-transcription polymerase chain reaction (RT-PCR) analysis was positive for severe acute respiratory syndrome coronavirus 2 (SARS-CoV-2). The patient did not respond to any treatment and died 13 days after admission. The possibility of COVID-19 in a patient must always be considered, especially in the current scenario, to prevent nosocomial infection from spreading.

## Introduction

The initial cases of severe acute respiratory syndrome coronavirus 2 (SARS-CoV-2) pneumonia occurred in Wuhan, Hubei Province, China, in December 2019 [1]. In Japan, the infection has continued to spread since the first confirmed diagnosis on January 15, 2020. By December 2021, the SARS-CoV-2 has spread all over the world. The total number of deaths due to SARS-CoV-2 has reached more than five million in the world, and nearly 20,000 people have died in Japan [2,3]. A variety of variants have emerged, and countries have been forced to deal with them. In this report, we present the clinical course and computed tomography (CT) findings of the first patient to die from coronavirus disease (COVID-19) pneumonia in Japan. Although our patient died, we prevented nosocomial infection by following appropriate infection control protocols without the use of a negative pressure chamber. We believe that this is an epidemiologically and clinically very important case that can show the situation in Japan at that time.

## Case presentation

**Figure 1:**
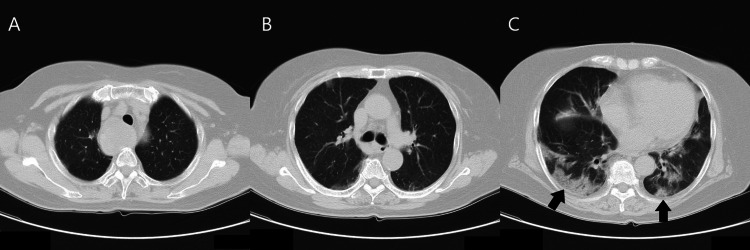
(A-C) Chest computed tomography images taken five days before being transferred to our hospital Peripheral ground-glass opacities are evident, predominantly in both lower lobes of the lungs.

An 82-year-old woman was admitted to a hospital with a seven-day history of fatigue and sudden development of fever and hypoxemia. The patient’s body temperature (BT) was 38.9℃, and oxygen saturation was 92% (room air) at that time. Chest CT showed peripheral ground-glass opacities (GGOs), predominantly in both lower lobes of the lungs (Figure 1). After five days of antibiotic treatment (ciprofloxacin), the patient was transferred to our hospital due to worsening oxygen saturation.

**Figure 2:**
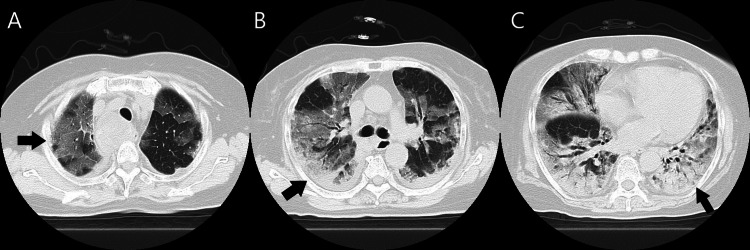
(A-C) Chest computed tomography images on day 1 Multiple bilateral ground-glass opacities are evident in all lobes, and consolidation is evident in both lower lobes of the lungs.

On presentation (on day 1 of admission to our hospital), BT was 37.0℃ without the use of antipyretics, blood pressure was 148/77 mmHg, respiratory rate was 38 breaths/min, and pulse was 89 beats/min. Oxygen saturation was approximately 90% using 6 L/min oxygen supplementation via a reservoir bag. Laboratory tests showed elevated levels of white blood cells (WBCs) and C-reactive protein (CRP) (Table 1), and chest CT showed rapid deterioration (Figure 2).

**Table 1:** Laboratory findings during the clinical course of the patient AST, Aspartate aminotransferase; ALT, alanine transaminase; CK, creatine kinase; CRP, C-reactive proteins; Hb, hemoglobin; LDH, lactate dehydrogenase; WBC, white blood cells.

Day	WBC, ×10^9^/L	Neutrophil, %	Hb, g/dL	Platelets, ×10^9^/L	CK, U/L	Creatinine, mg/dL	Albumin, g/dL	AST, U/L	ALT, U/L	LDH, U/L	CRP, mg/dL
5 days before being transferred to our hospital	4.64	62.3	12.4	146	87	0.5	4.1	95	67	336	5.7
3 days before being transferred to our hospital	4.62	70.1	12	140	67	0.5	3.6	88	59	389	5.3
Day 1	10.25	93	12.6	164	28	0.37	2.5	60	49	538	14.1
Day 2	8.53	92.8	12.6	179	20	0.39	2.4	39	39	464	14.64
Day 5	14.11	95	12.4	238	14	0.42	2.4	38	37	561	3.09
Day 7	23.92	98	13.9	192	30	0.42	2	28	18	919	28.01

The patient had never smoked and did not have any relevant medical history except for the mediastinal tumor.

Our patient neither had been to Wuhan nor had any direct contact with individuals that had been to or come back from Wuhan or were symptomatic with COVID-19. However, some individuals near the patient’s family were affected by pneumonia and showed signs of illness of unknown cause.

Taking these into consideration, we suspected that the patient might be infected with SARS-CoV-2. Thus, when the patient was transferred to our hospital, we asked the health center if we could perform a diagnostic COVID-19 reverse-transcription polymerase chain reaction (RT-PCR) assay. However, the center declined because she did not meet the definite criteria. In Japan at that time, all the criteria from one of the following options had to be met for COVID-19 testing via polymerase chain reaction: (1) BT ≥ 37.5℃, respiratory symptoms, and traveled to or resided in Hubei within 14 days before the onset of symptoms; (2) BT ≥ 37.5℃, respiratory symptoms, and had been in close contact with those who had traveled to or lived in Hubei within 14 days before the onset of symptoms; and (3) fever or respiratory symptoms and had been in close contact with patients with COVID-19.

Although we could not perform the diagnostic RT-PCR assay, an infectious disease, including COVID-19, was strongly suspected. Therefore, we admitted her to a private room as our hospital does not have a negative pressure room. All hospital staff that came in contact with the patient wore gloves, long-sleeved disposable gowns, protective eyewear, and surgical masks. In situations where the emission of aerosols was possible, the staff wore N95 masks.

**Figure 3:**
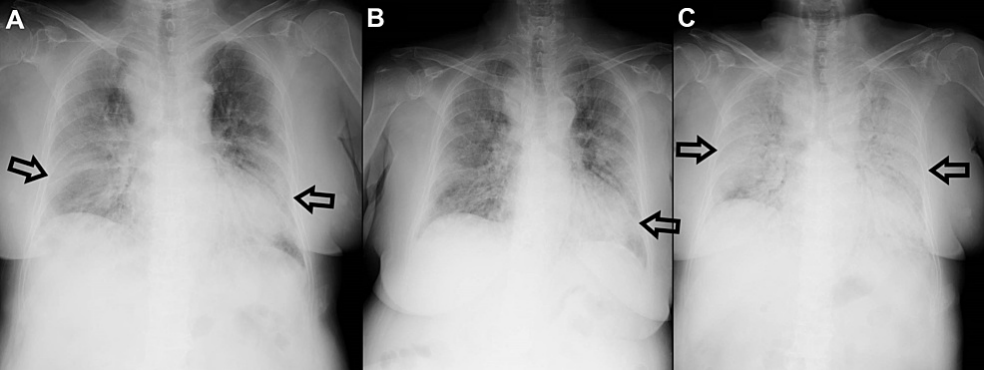
Chest radiographs (A) Day 2, (B) day 5, and (C) day 7.

After admission to our hospital, we initiated noninvasive positive pressure ventilation (NPPV) (because the patient did not consent for intubation) and administered the antibiotics meropenem (1.5 g/day) and levofloxacin (500 mg/day) as well as the antiviral peramivir (300 mg/day), and corticosteroids (1000 mg/day from day 1 to day 3, 40 mg/day after day 4). From the patient’s arterial blood gas results, we diagnosed acute respiratory distress syndrome (ARDS) and started sivelestat sodium hydrate. After starting these treatments, the patient’s subjective symptoms began to improve. Although her oxygen saturation and oxygen demand did not change, a chest x-ray showed an improvement in the shadow (Figure 3, Panel B). However, on day 6, the patient’s oxygen demand started to gradually increase from a fraction of inspired oxygen 0.6 to 1.0. After that, the patient did not respond to any treatment. A chest x-ray revealed a deterioration of the shadow (Figure 3, Panel C), and laboratory data showed elevated levels of WBC and CRP (Table 1) on day 7.

We again requested the health center for permission to perform a diagnostic COVID-19 RT-PCR assay. On day 7, we were granted permission to take a throat swab and sputum sample and submit them for RT-PCR. However, on day 8, the patient died. Following the patient’s death, the result was positive for SARS-CoV-2. After her death, written informed consent was obtained from the patient’s family to report her case. Moreover, all medical staff (31 personnel) who had been in contact with the patient were tested for COVID-19 via PCR analyses of pharyngeal swabs, and all results were negative. At that time, antibody tests for IgG and IgM had not been developed.

## Discussion

We report the first case of death due to COVID-19 in Japan. Moreover, we could prevent nosocomial infection because the possibility of COVID-19 infection was considered from the beginning.

Our patient had few symptoms. However, regarding the symptoms, 98.6% of patients have a fever, 44%-69.6% have fatigue, and 59.4%-76% have cough [4]. We suspect that this atypical COVID-19 clinical course was related to the patient’s age. It is well known that elderly patients are less symptomatic and afebrile [5]. On the other hand, CT findings were typical of COVID-19 pneumonia [6]. In our patient, GGOs were evident bilaterally in the lower lobes of the lungs. With a crazy-paving appearance, the GGOs extended to the upper lobes of the lungs and advanced to consolidation. More subpleural lesions were found than central lesions. It is estimated that 8%-29% of patients develop ARDS [1,7]. Even in patients with atypical symptoms, chest CT findings are characteristic of typical COVID-19 pneumonia, once their condition starts deteriorating.

The source of infection in our patient is not certain. However, a follow-up investigation by the health center revealed that the pneumonia outbreak in the individuals near the patient’s family was caused by SARS-CoV-2. We suspect this could be the source of infection in our patient.

In parallel with our case, COVID-19 patients unrelated to Wuhan nor had any contact with COVID-19 were detected in several areas of Japan. Thus, domestic community-acquired infection of COVID-19 was strongly suspected. A few days after our case, the criteria for COVID-19 testing had been relaxed, allowing testing for patients with BT ≥ 37.5℃, respiratory symptoms, and suspected pneumonia requiring hospitalization. This might have helped to clarify the infection situation in Japan.

Nosocomial infections with SARS-COV-2 have been reported in several hospitals [7,8], and it is said that 3.5% of the infections occurred in the medical staff [9]. In this case, it took a week to reach a diagnosis because of the domestic criteria for diagnostic COVID-19 PCR analysis. After the patient was confirmed with SARS-CoV-2 infection, all medical staff in contact with the patient were tested, and the results were negative.

We used NPPV in this case. NPPV is useful to support respiration, but it requires strict infection control because it can spread the virus to other individuals [10]. In this case, all the medical staff wore personal protective equipment [11,12]. After the patient’s death, the entire staff was monitored for two weeks [13], but none of them showed any signs of illness. Moreover, none of the other patients admitted to the same ward developed symptoms consistent with the clinical course. Hence, we excluded the possibility of nosocomial infection. Although there were no case reports, treatment guidelines, or infection control guidelines in Japan at the time, we successfully prevented nosocomial infection.

## Conclusions

We encountered the first death in Japan attributed to COVID-19 pneumonia using NPPV. In a hospital environment without a negative pressure chamber, following the appropriate infection control protocols can prevent nosocomial infections under the possibility of airborne transmission. We hope that the measures in this case contribute to the evidence of infection control against SARS-CoV-2.
